# The *Iroquois* Complex Is Required in the Dorsal Mesoderm to Ensure Normal Heart Development in *Drosophila*

**DOI:** 10.1371/journal.pone.0076498

**Published:** 2013-09-23

**Authors:** Zhasmine Mirzoyan, Petra Pandur

**Affiliations:** Institut für Biochemie und Molekulare Biologie, Universität Ulm, Ulm, Germany; University of Dayton, United States of America

## Abstract

*Drosophila* heart development is an invaluable system to study the orchestrated action of numerous factors that govern cardiogenesis. Cardiac progenitors arise within specific dorsal mesodermal regions that are under the influence of temporally coordinated actions of multiple signaling pathways. The *Drosophila Iroquois* complex (*Iro-C*) consists of the three homeobox transcription factors *araucan* (*ara*), *caupolican (caup*) and *mirror (mirr*). The *Iro-C* has been shown to be involved in tissue patterning leading to the differentiation of specific structures, such as the lateral notum and dorsal head structures and in establishing the dorsal-ventral border of the eye. A function for *Iro-C* in cardiogenesis has not been investigated yet. Our data demonstrate that loss of the whole *Iro* complex, as well as loss of either *ara/caup* or *mirr* only, affect heart development in *Drosophila* . Furthermore, the data indicate that the GATA factor Pannier requires the presence of *Iro-C* to function in cardiogenesis. Furthermore, a detailed expression pattern analysis of the members of the *Iro-C* revealed the presence of a possibly novel subpopulation of Even-skipped expressing pericardial cells and seven pairs of heart-associated cells that have not been described before. Taken together, this work introduces *Iro-C* as a new set of transcription factors that are required for normal development of the heart. As the members of the *Iro-C* may function, at least partly, as competence factors in the dorsal mesoderm, our results are fundamental for future studies aiming to decipher the regulatory interactions between factors that determine different cell fates in the dorsal mesoderm.

## Introduction

One of the fundamental questions in developmental biology is how multiple recurring signal inputs are interpreted and integrated to generate different cell fates. The dorsal mesoderm of *Drosophila* embryos is an ideal system to study the molecular mechanisms that determine the different developmental fates of dorsal mesodermal cells. The dorsal mesoderm initially becomes subdivided into the cardiac, visceral and somatic mesodermal domains, which harbor progenitors for the heart (myocardial and pericardial cells), the circular midgut muscles and for some somatic, dorsolateral body-wall muscles [1]. The determination of these three mesodermal primordia, as well as the specification and differentiation of the different mesodermal cell types require complex interactions between a number of factors including signaling pathways initiated by the Wnt family member Wingless (Wg), the TGF-β family member Decapentaplegic (Dpp), the Notch signaling pathway and the Ras/mitogen-activated protein kinase (MAPK) pathway, which can be activated by the epidermal (EGFR) or fibroblast growth factor receptor (FGFR) [2,3,4,5,6,7,8]. Previous studies have established a hierarchy of these pathways where the ectodermal Wg and Dpp signals prepattern the mesoderm. *Dpp* is expressed along the dorsal edge of the ectoderm and its signal is required for the induction of the visceral mesoderm [4,9]. Dpp maintains the mesodermal expression of tinman (tin), a homeobox transcription factor crucial for rendering cells competent to respond to the signaling pathways mentioned above [4,10]. *Wg* is expressed in stripes perpendicular to *dpp* and is restricted to the anterior domain of each trunk segment. The combined activity of Dpp and Wg distinguishes the cardiac and somatic mesoderm from visceral mesoderm [11,12]. *Tin* is indispensable but not sufficient for the formation of the different dorsal mesodermal primordia [10,13,14]. In addition to *tin*, cardiac mesoderm formation depends on the GATA factor *pannier* (*pnr*), the T-box factors *Dorsocross1-3* (referred to as *Doc*) and the LIM-homeodomain transcription factor *tailup* (*tup*) [15,16,17,18,19]. Once the cardiac progenitors have been specified they differentiate into myocardial and pericardial cells that build the fly heart.

In *Drosophila* , the *Iroquois* complex (*Iro-C*) of homeobox transcription factors consists of three highly conserved TALE (three amino acid loop extension) homeodomain proteins Araucan (Ara), Caupolican (Caup) and Mirror (Mirr). In addition to the conserved homeodomain, the amino acid sequence of Iro-C members harbors an EGF-like motif [20,21,22], which represents a putative protein-protein interaction domain that is similar to the interaction domain of Notch proteins [20,21]. Two members, Ara and Caup, also contain two putative MAPK phosphorylation sites, suggesting that their transcriptional activity can be regulated by the MAPK pathway [20]. Indeed, such a regulation has been described for Caup [23] and for vertebrate Irx2 [24]. Alignments of the three proteins show that Ara and Caup are more similar to each other than they are to Mirr, however, all three proteins share a high sequence identity in the homeodomain and in the EGF-like motif [20,21]. Furthermore, Ara and Caup have highly similar expression patterns and were shown to function redundantly in different tissues [20,22,23]. Previous studies have identified two distinct functions for *Iro-C*. First, *Iro-C* is involved in establishing domains in which the cells acquire competence to properly interpret and respond to the signaling pathways they are exposed to, hence the cells acquire a particular differentiation potential. This has been studied with respect to notum patterning, specification of dorsal head structures and the dorsal-ventral subdivision of the eye [25,26,27,28,29]. Second, *Iro-C* is required for the specification and/or differentiation of specific structures or cell types such as wing veins, sensory organs of the notum and some lateral transverse muscles [20,23]. In vertebrates, the *Iro* complex comprises six members (*irx1-6*) that are expressed both in distinct and overlapping domains including the developing mammalian heart [30,31]. Owing to their expression in specific structures of the heart and possible functional redundancy, the overall heart phenotypes of single *irx* knockout mice are not dramatic, yet a missing *irx* gene does impact proper heart function [31]. For example, *irx3* is required for the normal electrophysiological properties of the heart [32] and adult *irx4*-deficient mice develop cardiomyopathy [33].

We were interested to determine whether *Iro-C* functions in *Drosophila* heart development since this process also requires the establishment of a domain that is competent to respond to cardiac signals. To this end we investigated the expression pattern of Ara/Caup and Mirr during embryogenesis and analyzed the heart phenotypes in different *Iro-C* mutant embryos. At early embryonic stages, the expression pattern suggests an involvement of *Iro-C* in patterning the dorsal mesoderm along the anterior-posterior (AP) axis. Analyses of cardiac markers in different *Iro-C* mutants at early stages confirmed this hypothesis. Interestingly, the expression of all crucial cardiac transcription factors was affected except for the GATA factor *pnr*. Analyses of heart markers at late embryonic stages revealed distinct phenotypes showing a disorder in the stereotypic pattern of the heart cells. By stage 15 we detected the expression of Ara/Caup in the most anterior Even-skipped (Eve) pericardial cells. Interestingly, we also observed Ara/Caup in cells distributed evenly along the heart tube that appear to be distinct from any of the heart cells known so far. These newly identified Ara/Caup positive heart-associated cells also expressed the third Iro-C member, Mirr. Mirr protein was additionally expressed in a cell adjacent to the Ara/Caup/Mirr-positive cell along the heart tube. Taken together, our data describe the cardiac mesoderm as an additional domain where members of the *Iro-C* may function as competence factors, which are also involved in the differentiation of heart cell types.

## Materials and Methods

### *Drosophila* *stocks* and crosses

The following mutant fly stocks were used: *Df*(*3L*)*iro-2* (stock# 4507), *Df*(*3L*)*iro* ^DFM3^ (stock# 36531), *tup* ^*isl-1*^ (stock# 3556), *pnr* ^VX6^ (stock# 6334) (all from The Bloomington Stock Center), *mirr* ^*e48*^ [21], *iro* ^DFM1^ [20], *tin* ^346^ [13] and *Df*(*3L*)*DocA* [34]. *Df*(*3L*)*iro-2*, *Df*(*3L*)*iro* ^DFM3^, *mirr* ^*e48*^ and *iro* ^DFM1^ flies were rebalanced with *TM3, ftz-lacZ* to identify homozygous mutant embryos. CantonS (stock#1) served as a wild-type stock. Of note, the deficiency *Df*(*3L*)*iro-2* lacks several other genes in addition to *ara*, *caup* and *mirr*. Although none of these genes have been associated with heart development, a few of them are involved in modulating different signaling pathways, e.g. the Wg and Notch pathways. The following Gal4 and UAS-lines were used: *twi*-Gal4;*24B*-Gal4 [17], UAS-*caup*, UAS-*ara* [20], UAS-*mirr* [21].

### Immunohistochemistry and in situ hybridization

Antibody staining (single and double labeling) was performed essentially as described [35]. Primary antibodies that did not require amplification of the signal were detected with a DyLight594-conjugated AffiniPure donkey anti-rabbit IgG (H+L) (1:200) (Dianova, Hamburg, Germany). If amplification of the signal was necessary, biotinylated secondary antibodies were used (1:200) (Dianova) in combination with the Tyramide Signal Amplification System (Perkin Elmer, Rodgau, Germany) and dichlorotriazinylamino fluorescin (DTAF) (1:200) (Dianova). Embryos were mounted in Vectashield (Vector Laboratories). Embryos from immunostainings and *in situ* hybridizations were analyzed using the Keyence BZ-8000K epifluorescence microscope, with the image-analyzing software BZ-Analyzer (Keyence, Neu-Isenburg, Germany) and on the Leica TCS SP5II confocal microscope. Statistical computing was performed using GraphPad Prism (Prism-graphpad.com).

Primary antibodies were used at the following dilutions: rat anti-Caup (1:200), this antibody recognizes Ara as well [36,37], rabbit anti-Mirr (1:500) [38], rabbit anti-Dmef2 (1: 2000) [39], rabbit anti-Tin (1:500) [40], rabbit anti-Tin (1:1000) [41], rabbit anti-β3Tubulin (1:5000) [42], rabbit anti-Odd (1:100) [43], rabbit anti-Doc 2 (1:2000) [34], rabbit anti-Eve (1:1000) [44], rabbit anti-Zfh-1 (1:5000) [45]. The following primary antibodies were from the Developmental Studies Hybridoma Bank (DSHB): mouse anti-En (1:1), mouse anti-Wg (1:1), mouse anti-chicken Isl1 (Tup) (1:20) [19], mouse anti-Eve (1:20), mouse anti-FasIII (1:100), mouse anti-Prc (EC11) (1:20).

Fluorescent *in situ* hybridization for *caup*, *dpp* and *pnr* was performed according to standard procedures. The *dpp* antisense RNA was generated from the 2.9 kb *dpp* E55 fragment [46], the *pnr* antisense RNA from a 2.6 kb fragment [47]. The plasmid containing *caup* cDNA (RE64213) was obtained from the *Drosophila* Genomics Resource Center (DGRC). For generating an *in situ* RNA probe, a 982 bp fragment of the *caup* cDNA (EcoRI / HindIII) was subcloned into pBKS. Double fluorescent *in situ* hybridization and immunostaining was adapted from Knirr et al. (1999) [48]. The digoxigenin-labeled RNA *in situ* probes were generated using the DIG RNA Labeling mix from Roche (Mannheim, Germany).

## Results

### Embryonic expression pattern of the members of the Iro-C

**Figure 1:**
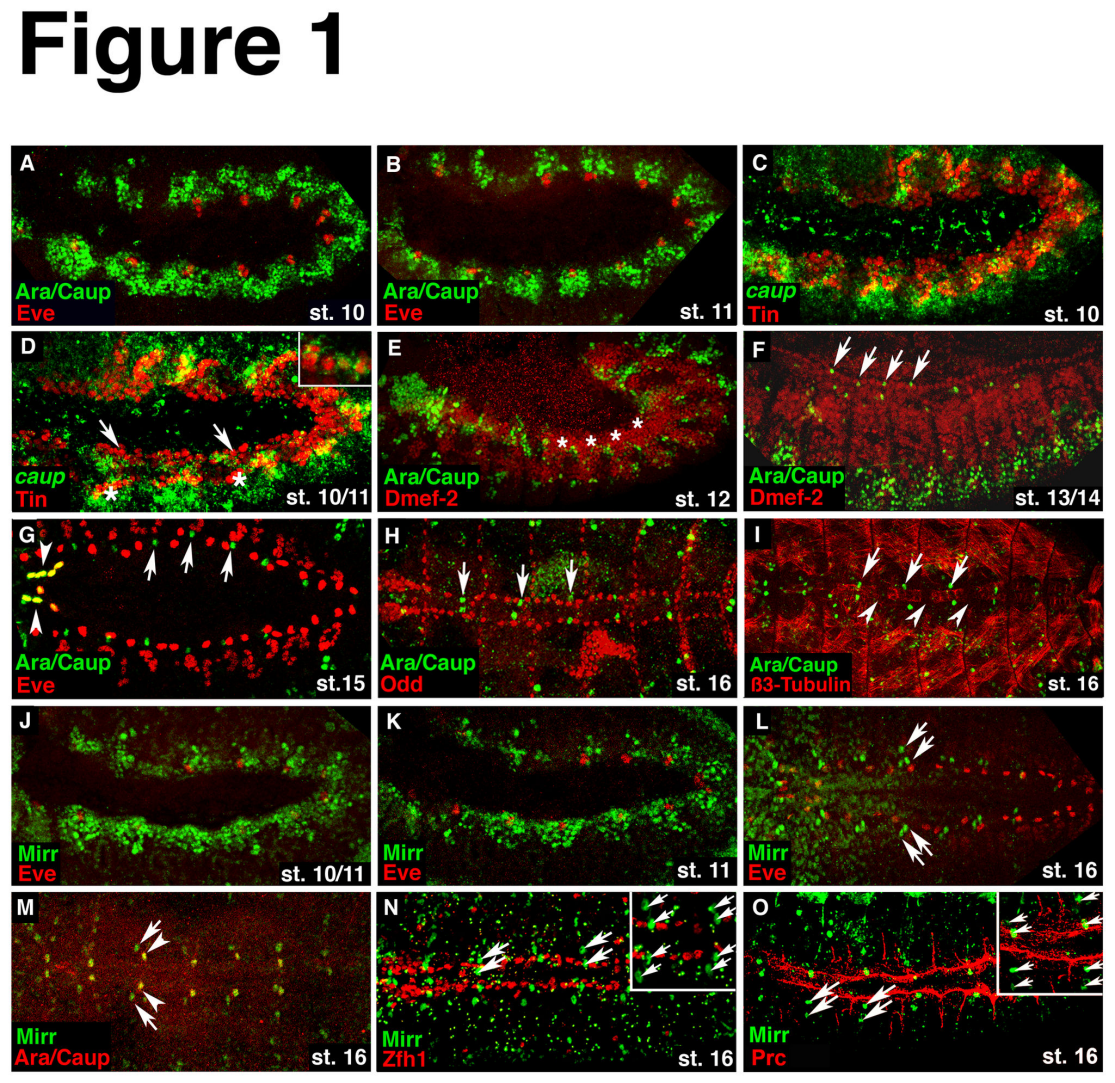
Expression pattern of Ara/Caup and Mirr during embryogenesis in wild-type embryos. (A) Double immunostaining for Ara/Caup and Eve shows the mesodermal expression of Ara/Caup as a continuous band along the dorsal side of the embryo. (B) During stage 11 Ara/Caup proteins are present in segmental clusters abutting the Eve cell clusters. (C) *In* *situ* hybridization for *caup* transcripts and immunostaining for Tin further demonstrates the presence of *caup* in the dorsal mesoderm. (D) Starting at stage 11 Tin-positive cells segregate into cardiac cells (arrows) and cells of the visceral mesoderm (asterisks). There is still a considerable overlap of Tin and *caup* mRNA expression. The inset in (D) show a higher magnification to better visualize *caup* mRNA transcripts around Tin protein that is located in the nucleus. (E) By stage 12 Ara/Caup expression has vanished from the dorsal mesoderm (asterisks) where heart cells begin to differentiate. (F) Starting at mid-embryogenesis, single Ara/Caup-positive cells arise along the Dmef2 expressing myocardial cell row (arrows). (G) Double labeling for Ara/Caup and Eve at stage 15 shows their co-expression in the most anterior Eve-positive pericardial cells (arrowheads), as well as Ara/Caup-only expressing cells located between the Eve pericardial cells (arrows). (H) The Ara/Caup-expressing cells (arrows) are not positive for the pericardial cell marker Odd. (I) β3-Tubulin that labels the four Tin-positive myocardial cells in each hemisegment by stage16 and allows a more accurate localization of the Ara/Caup expressing cells (arrows) along the heart tube. The arrowheads point to the β3-Tubulin-negative myocardial cells in each segment. These cells express Seven-up and Doc. (J) Mirr expression in the dorsal mesoderm is similar to the expression of Ara/Caup at early embryonic stages. (K) During stage 11, Mirr protein accumulates around the Eve-positive cell clusters. (L) At stage 16, pairs of Mirr-only expressing cells are detected between the Eve pericardial cells (arrows). (M) Double labeling for Ara/Caup and Mirr reveals the co-expression of all three factors in one cell (arrowheads) of the segmentally arranged Mirr expressing (arrows) cell pairs. (N) None of the Zfh-1-expressing pericardial cells co-expresses Mirr. (O) Double immunostaining for Mirr (arrows) and the extracellular matrix molecule Pericardin (Prc) that labels pericardial cells and is expressed along the extensions of the alary muscles, shows that the Mirr-positive cells are located between these extensions. Additionally, one of the two Mirr-positive cells lies adjacent to the Prc-expressing basal membrane of the myocardial cells. All embryos are oriented with the anterior to the left. Embryos in A-F, J, K are shown from the lateral side. A dorsal view of the embryos in G, H, I, L-O is shown.

We first evaluated the expression pattern of Ara/Caup and Mirr with respect to their possible involvement in heart development. Of note, since the only available and widely used antibody against Caup also detects Ara, the immunostainings are labeled as Ara/Caup [23,36,37]. At stage 10 Ara/Caup-expressing cells are detected along the dorsal edge of the ectoderm (data not shown and [27,37]) and in an undulating pattern in the dorsal mesoderm (Figure 1A). During stage 11 the initially continuous expression of Ara/Caup in the dorsal mesoderm becomes restricted to segmental patches with the Ara/Caup-positive clusters abutting the Eve expressing cells that are progenitors for pericardial cells and dorsal somatic muscles [44,49] (Figure 1B). An *in situ* hybridization for *caup* mRNA transcripts coupled with an immunostaining for Tin confirms the presence of *caup* mRNA in the dorsal mesoderm that encompasses cardiac and visceral mesoderm (Figure 1C). *Ara* mRNA transcripts are detected in a similar pattern albeit at a lower level compared to the mesodermal expression of *caup* mRNA transcripts (data not shown). When Tin expression becomes restricted to dorsal and ventral clusters that represent cardiac and visceral mesodermal cells, respectively, we detect a stronger expression of *caup* mRNA around the ventral clusters of Tin-positive cells (Figure 1D). During stage 12, Ara/Caup-positive cells disappear in the dorsal region where heart progenitors have been specified and begin to differentiate (Figure 1E). During stage 13 we detect single Ara/Caup-expressing cells alongside the row of Dmef2-positive myocardial cells (Figure 1F). A double immunostaining for Ara/Caup and the pericardial cell marker Eve shows that the Ara/Caup-expressing cells are located between the Eve pericardial cells by stage 15. The Eve pericardial cells at the anterior tip of the heart co-express Ara/Caup (Figure 1G). To determine whether the Ara/Caup-positive cells that appear to be embedded between the Eve pericardial cells belong to one of the known pericardial cell types, we performed double immunostainings for Ara/Caup and Odd (Figure 1H), Ara/Caup and Tin (Figure S1A) and a double staining for *caup* mRNA and Zfh-1 protein (data not shown). We did not detect a co-expression of Ara/Caup with any of these factors. A double immunostaining for Ara/Caup and the structural protein β-Tubulin, a marker that specifically labels the four Tin-positive myocardial cells in each hemisegment [50], shows that the Ara/Caup-expressing cells are located laterally to the β-Tubulin-expressing (and therefore also Tin-positive) cells (Figure 1I). The expression pattern of Mirr is similar to that of Ara/Caup in that Mirr protein is detected in the dorsal mesoderm by stage 10/11 with a more pronounced expression around the Eve cell clusters (Figure 1J) and in the dorsal ectoderm (data not shown and [21]). Similar to Ara/Caup, the initial continuous expression along the dorsal side becomes restricted to segmental patches during stage 11 (Figure 1K) and vanishes from the dorsal mesoderm during mid-embryogenesis (data not shown). By stage 15/16 Mirr is expressed segmentally in pairs of cells along the heart tube, however, unlike Ara/Caup, Mirr is not expressed in any of the Eve pericardial cells (Figure 1L). Moreover, one of the two Mirr positive cells co-expresses Ara/Caup (Figure 1M). From this observation we conclude that the Ara/Caup expressing cells embedded between the Eve pericardial cells shown in Figure 1G are also positive for Mirr. We did not observe a co-localization of Mirr and Zfh-1 (Figure 1N) or of Mirr and Tin (Figure S1B) in pericardial cells. To obtain more information on the localization of the Mirr-expressing cell pairs, we performed a double-immunostaining for Mirr and the pericardial cell marker, Pericardin (Prc) (Figure 1O). Prc is an extracellular matrix protein that is detected around pericardial cells and that strongly labels the basal membrane of the epithelial myocardial cells [51]. Additionally, Prc is expressed along the seven alary muscles projecting from the dorsal vessel. The Mirr-expressing cell pairs lie approximately in the middle between the Prc-positive extensions with one cell being almost adjacent to the Prc-expressing basal membrane of the myocardial cells. In summary, the early expression pattern of members of the *Iro-C* in the dorsal mesoderm suggests a role for these factors in establishing territories with different cell fates. The co-expression of Ara/Caup and Eve in the anterior Eve pericardial cells suggests a function for Ara/Caup in the diversification of pericardial cells. Lastly, the Ara/Caup/Mirr-positive cells, as well as the Mirr-only positive cells detected along the forming heart tube may represent novel heart-associated cells.

### Loss of Iro-C affects expression of crucial cardiac transcription factors except for pnr

The early expression pattern of the *Iro-C* factors suggests that they are involved in patterning the dorsal mesoderm thereby ensuring the normal development of its derivatives. In the present study we wanted to determine the impact of the loss of *Iro-C* on heart development. We initially analyzed embryos that contain a deletion on the third chromosome (*Df(3L*)*iro-2*) that eliminates all three members of the *Iro-C* (*ara*, *caup* and *mirr*) [20,52,53]. The phenotypes of an additional deficiency line that lacks *ara*, *caup* and *mirr* (*iro* ^*DFM3*^) [36] are shown in Figure S2. Since Ara and Caup have been indicated to act redundantly in different tissues, we chose a second mutant that lacks *ara* and *caup* but still expresses *mirr* (*iro* ^*DFM1*^) [20,53,54]. To validate the contribution of *ara* and *caup* to the observed heart phenotypes we analyzed embryos with the genetic background *Df*(*3L*)*iro-2*/*iro* ^*DFM1*^. These embryos are homozygous mutant for *ara* and *caup* and heterozygous for *mirr*. Since *mirr* is more divergent from *ara* and *caup* and could either not play a role in cardiogenesis at all or elicit a different heart phenotype we investigated embryos mutant for *mirr* (*mirr* ^*e48*^) separately. The *mirr* ^*e48*^ allele contains a 1kb deletion in the *mirr* promoter region and therefore *mirr* ^*e48*^ mutants are devoid of the gene product [21,55].

**Figure 2:**
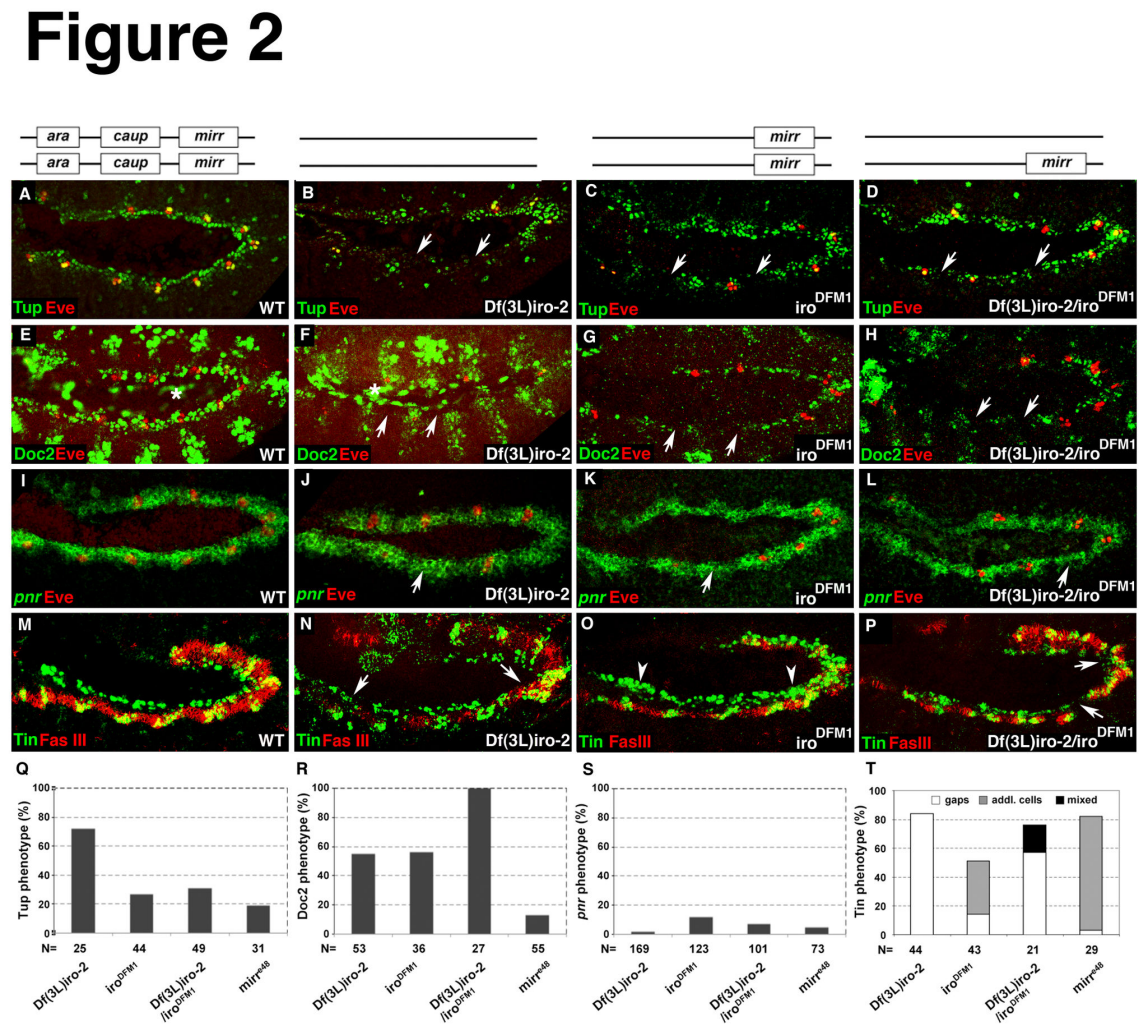
*Iro-C* is required for the normal expression of early cardiac transcription factors. Phenotypes were analyzed in different *Iro-C* mutants. The genetic background is depicted in the drawing above each column. The boxes illustrate the presence of the indicated factor. (A-D). Embryos mutant for *Iro-C* exhibit a reduction of Tup-expressing cells (arrows). (E-H) Doc 2- expressing cells are strongly reduced along the dorsal edge of the mesoderm (arrows). Asterisks in E and F indicate Doc 2 expression in the amnioserosa. (I-L) *pnr* mRNA expression is unaffected in 85-95% of the different *Iro-C* mutants. Arrows point to missing Eve cell clusters. (M-P) Tin expression is affected in the different *Iro-C* mutants. Images are merged optical sections of double immunostainings for Tin and Fasciclin III (FasIII) in which Tin-positive cells in the visceral mesoderm labeled by FasIII appear yellow. (N) *Df*(3L)*iro-2* embryos are characterized by a reduction of Tin-expressing cells (arrows) and reduced FasIII expression. (O) Embryos mutant for *ara/caup* only (iro^DFM1^) are characterized by an increase in Tin-positive cells in the cardiac region (arrowheads). (P) The predominant phenotype in *Df*(3L)*iro-2/* *iro* ^DFM1^ heterozygotes is a slight reduction of Tin and FasIII expression (arrows). (Q-T) Quantification of the phenotypes observed. The bar graphs depict the percentage of embryos that exhibited a phenotype. The bar graphs in Q-S depict the percentage of embryos that had reduced expression of the indicated markers. All images show lateral views of stage10/11 embryos; anterior is to the left.

**Figure 3:**
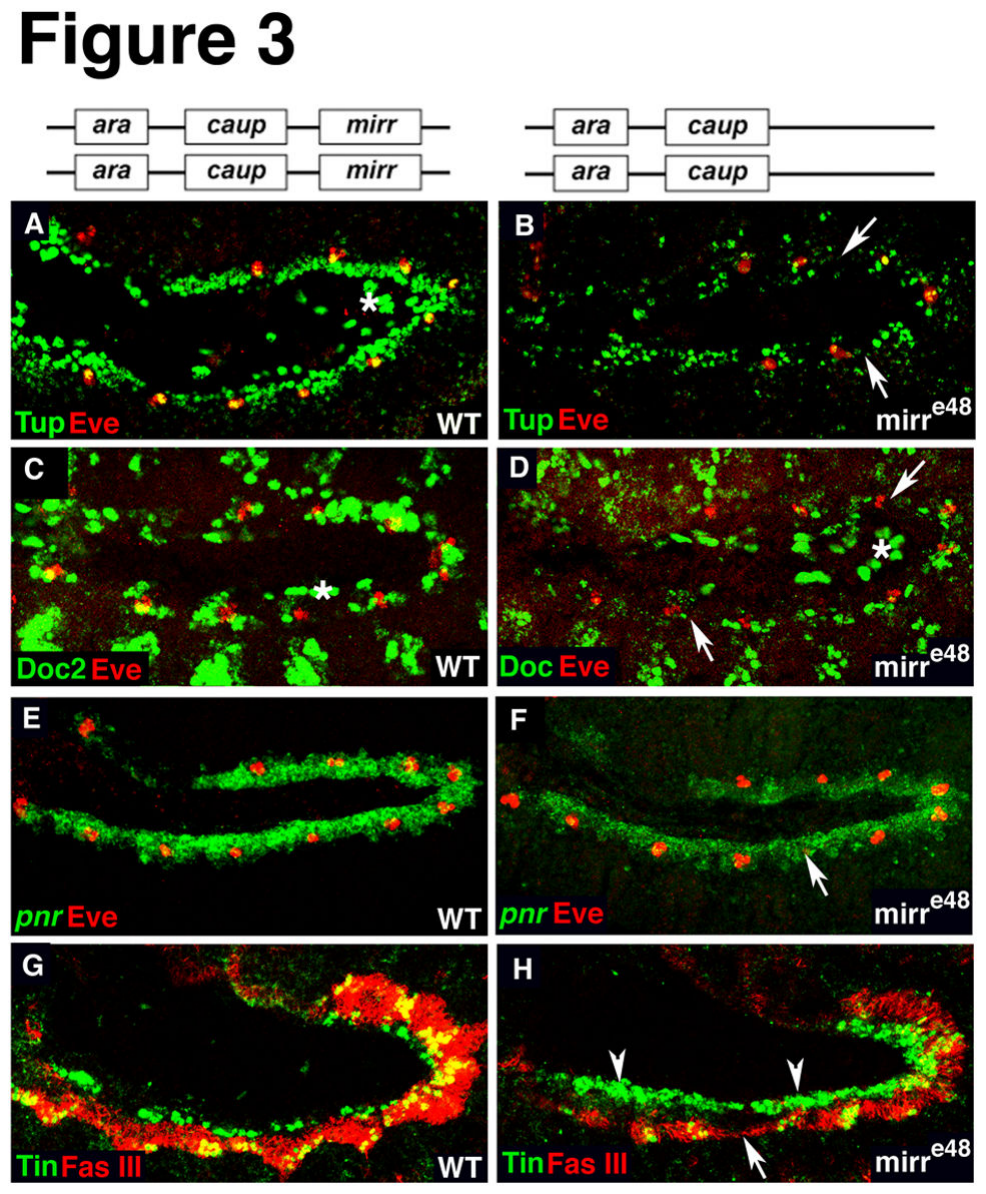
Loss of *mirr* affects the normal expression of early cardiac transcription factors. (A-D) *mirr* mutants are characterized by a reduction of (B) Tup- and (D) Doc-expressing cells (arrows). Asterisks in A, C and D indicate expression of Tup or Doc 2 in the amnioserosa. (E, F) *pnr* mRNA expression is not affected in *mirr* mutants. The arrow in F points to a missing Eve cell cluster. (G, H) Images are merged optical sections of double immunostainings for FasIII and Tin-expressing cells that appear yellow in the visceral mesoderm. *mirr* mutants exhibit an increase in Tin-expressing cells in the cardiac region (arrowheads). FasIII expression is slightly reduced in some embryos (arrow). All images show lateral views of stage10/11 embryos. The genetic background is depicted in the drawing above each column.

**Figure 4:**
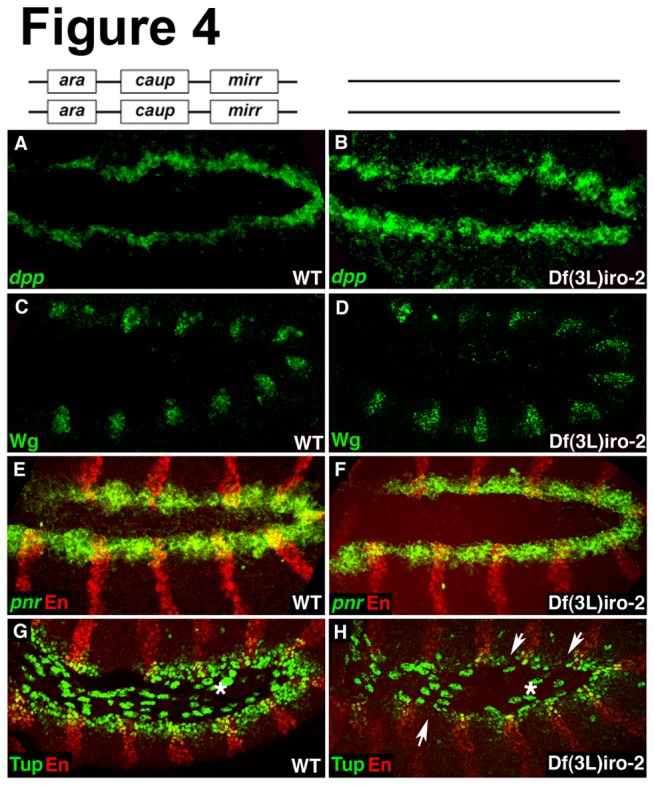
Wg and *dpp* expression in the ectoderm is independent of *Iro-C*. (A-D) Expression of the two crucial cardiac growth factors secreted from the ectoderm, *dpp* (A, B) and Wg (C, D) is unaffected in *Iro-C* mutants at early stages. (E, F) Ectodermal *pnr* mRNA expression does not depend on *Iro-C*. (G, H) *Iro-C* is required for ectodermal Tup expression (arrows). Asterisks in G and H indicate Tup expression in the amnioserosa. Images in E-H are confocal images of double stainings using Engrailed (En) as a marker for the ectodermal layer.

The transcription factors *tin*, *pnr*, *tup*, and *Doc* are well-characterized components of the transcriptional network that is crucial for the formation of the cardiac mesoderm and are required for the specification and differentiation of heart progenitors. To determine whether their expression depends on *Iro-C*, we analyzed the expression of Tup, Doc and Tin protein, as well as of *pnr* mRNA in the different *Iro* mutant genetic backgrounds at early stages. The bar graphs in Figure 2Q-T depict the percentage of embryos that exhibited a phenotype. The graphs include the quantification of heart phenotypes observed in *mirr* mutants shown in Figure 3. For both Tup and Doc, we observed a strong downregulation in the dorsal margin of the mesoderm, which corresponds to the cardiac region (Figure 2A-H and Figure 3A-D). A comparison of the penetrance of the phenotypes shows that unlike Tup, Doc is more strongly affected in mutants that lack *ara* and *caup* (*iro* ^*DFM1*^) (Figure 2Q, R). Since Doc 2 was downregulated only in 13% of the embryos mutant for *mirr*, it seems that *mirr* is less important for maintaining Doc (Figure 2R and Figure 3C, D). The expression of *pnr* transcripts was unaffected in the majority (85-95%) of embryos of all investigated mutants (Figure 2I-L, S and Figure 3E, F). Our current understanding of *pnr* function in the dorsal mesoderm is that *pnr* is required for maintaining Doc and Tup expression [18,19]. Since we observed a downregulation of Doc 2 in almost 60% of the *iro* ^DFM1^ embryos, this finding indicates that *ara*/*caup* are required in addition to Pnr to regulate Doc 2 expression. Analyses of Tin expression revealed that the majority of embryos mutant either for *ara/caup* or *mirr* exhibit an increased number of Tin-expressing cells (Figure 2M, O, T and Figure 3G, H). *Df*(*3L*)*iro-2* embryos lacking all three *Iro-C* members were characterized by small gaps in the Tin-expressing domains at stages 11/12 (Figure 2M, N, T). It may be that in this particular deficiency additional genes are deleted, which influences the phenotype. In fact, 76% of the *iro* ^DFM3^ mutant embryos that carry a smaller deletion and lack expression of all three *Iro-C* factors were also characterized by additional Tin-positive cells at stages 11/12 (Figure S2D, E). The majority of heterozygous *Df*(*3L*)*iro-2*/*iro* ^*DFM1*^ embryos showed a mild reduction of Tin and approximately 20% of the embryos showed a mixed phenotype consisting of small gaps and accumulation of Tin-positive cells (Figure 2M, P, T). It should be noted that the expression levels of *ara*, *caup* and *mirr* can differ in different *Iro-C* mutant genetic backgrounds [29]. Different expression levels of individual members of the *Iro-C* could influence the phenotype since Iro-C proteins were shown to act as homo- and heterodimers [56]. Hence, the proper stoichiometry can be disturbed to various extents in the different mutants. Embryos that only lack *mirr* are almost exclusively characterized by an overproduction of Tin-expressing cells (Figure 2T and Figure 3G, H). Tin is expressed in cardiac progenitors as well as in precursor cells of the visceral mesoderm (Figure 1D asterisks). Since Ara/Caup expression encompasses the visceral mesoderm we performed a double immunostaining for Tin and Fasciclin III (FasIII) to also label visceral mesodermal cells. FasIII and Tin expression was reduced to variable extents in the majority of *Df*(*3L*)*iro-2* embryos (Figure 2M, N), in *iro* ^DFM1^ mutant embryos (Figure 2M, O) and in embryos with the genetic background *Df*(*3L*)*iro-2*/*iro* ^*DFM1*^ (Figure 2M, P). In contrast to these mutants, the phenotype for FasIII was striking in *iro* ^DFM3^ mutants. 88% of the *iro* ^DFM3^ embryos (n =25) exhibited a strong reduction in FasIII expression (Figure S2E) and the remaining 12% were devoid of FasIII. Since the *Iro-C* factors are not only expressed in the mesoderm but also in the ectoderm ( [27,37] and data not shown), we tested whether *Iro-C* regulates the two ectodermal factors, Dpp and Wg that are crucial for heart development. If the expression of Dpp and/or Wg is affected in *Iro-C* mutants, this could also account for or contribute to the observed heart phenotypes. We did not detect changes in expression of *dpp* mRNA or Wg protein (Figure 4A-D). Consistent with our findings in the mesodermal layer, expression of *pnr* in the ectoderm is independent of *Iro-C* (Figure 4E, F) whereas ectodermal Tup expression requires *Iro-C* (Figure 4G, H). Taken together these findings indicate a role for *Iro-C* in the correct patterning of the dorsal mesoderm and in the differentiation of its derivatives.

### Embryos mutant for tin, pnr, Doc or tup show reduced expression of the Iro-C

**Figure 5:**
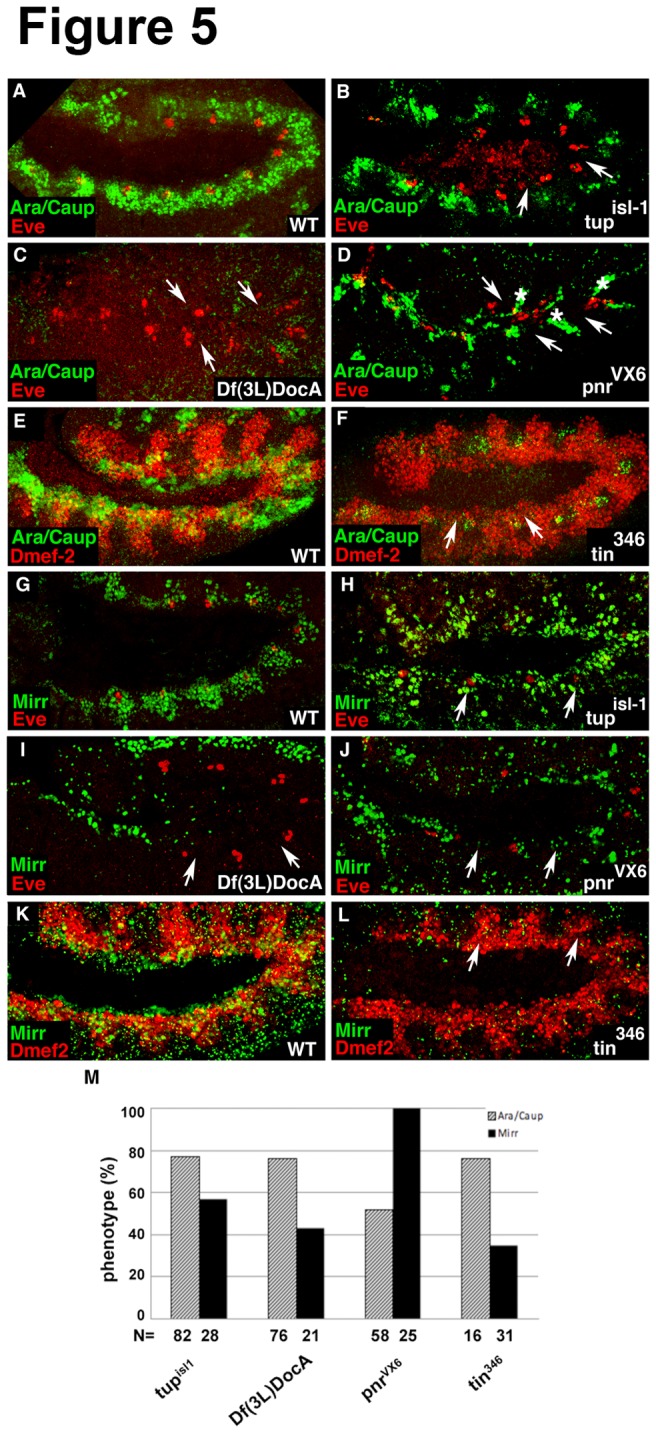
Mesodermal expression of Ara/Caup and Mirr depends on the crucial cardiac transcription factors *tup*, *Doc*, *pnr* and *tin*. (A, B) Ara/Caup expression is strongly reduced at the dorsal edge of the mesoderm (arrows) in *tup* ^*isl-1*^ mutants. (C) *Doc* mutants (Df(3L)DocA) show a dramatic loss of Ara/Caup expression in the dorsal mesoderm (arrows). (D) In *pnr* ^VX6^ mutants, Ara/Caup expression is severely reduced (arrows), but some Ara/Caup-positive cells remain in a striped pattern along the dorsal side of the embryo (asterisks). (E, F) A double immunostaining for Ara/Caup and Dmef2 reveals the absence of Ara/Caup in *tin* mutants (arrows). (G-J) Mirr-expressing cells are mildly reduced in *tup* ^*isl-1*^ mutants (arrows in H) whereas a strong reduction of Mirr-positive cells is observed in *Doc* mutants (Df(3L)DocA) (arrows in I) and in *pnr* ^VX6^ mutants (arrows in J). (K, L) *tin* mutants show a strong reduction of Mirr-expressing cells in the dorsal mesoderm (arrows in L). (M) Quantification of the phenotypes. The bar graph shows the percentage of embryos that were characterized by a reduction either of Ara/Caup or Mirr. Lateral views of stage 10/11 embryos are shown.

Having shown that except for *pnr*, all crucial cardiac factors are affected in embryos mutant for either the whole *Iro-C*, for *ara/caup* or *mirr* we were interested to determine whether *tup*, *Doc*, *pnr* and *tin* are required for the expression of Ara/Caup and Mirr. Indeed, the expression of Ara/Caup depends on *tup*, *Doc*, *pnr* and *tin* with the most dramatic loss seen in *tin* (*tin* ^*346*^) and *Doc* (*Df(3L*)*DocA*) mutants (Figure 5A, C, E, F). Embryos mutant for *tup* (*tup* ^*isl-1*^) exhibit a strong reduction of Ara/Caup-expressing cells (Figure 5A, B). The *pnr* (*pnr* ^*VX6*^) mutants were also characterized by a strong loss of Ara/Caup-expressing cells, however, we frequently observed residual Ara/Caup-positive cells in a segmentally arranged “stripe-like” pattern along the trunk (Figure 5A, D). In contrast to Ara/Caup, Mirr expression appears not as strongly reduced in *tup* mutants (Figure 5G, H, compare with B). Initiation and/or maintenance of Mirr expression requires *Doc*, *pnr* and *tin* since these mutants are characterized by a dramatic reduction of Mirr-expressing cells (Figure 5I, J, K, L). The quantification of the phenotypes is illustrated in Figure 5M, which shows the percentage of *tup*, *Doc*, *pnr* and *tin* mutant embryos in which the expression of Ara/Caup and Mirr was downregulated.

### Loss of Iro-C affects normal heart cell diversification and formation of the heart tube

**Figure 6:**
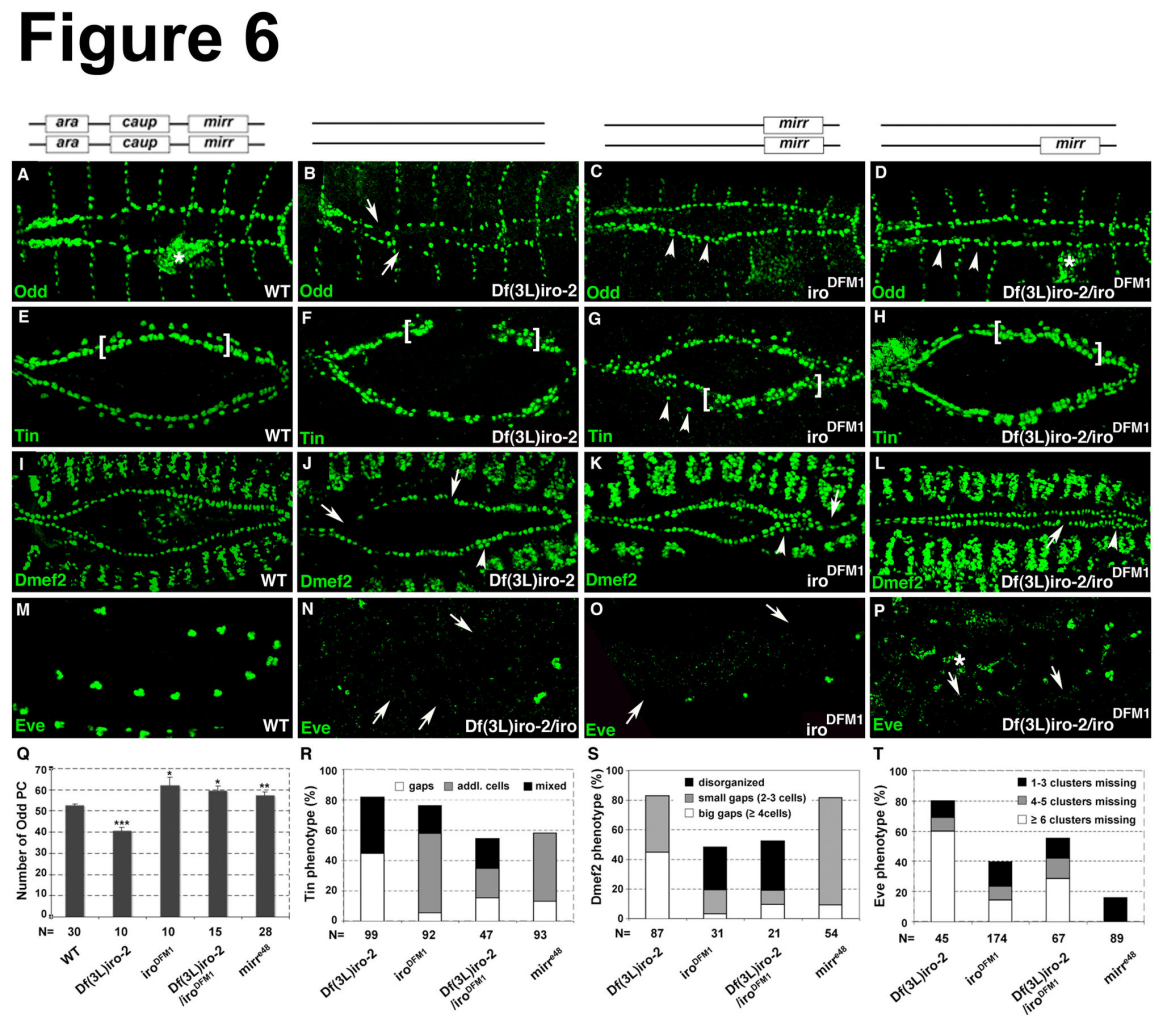
Heart phenotypes embryos mutant for *Iro-C*. The genetic background is depicted in the drawing above each column. The boxes illustrate the presence of the indicated factor. (A-D, Q) Loss of *ara/caup* results in an increase in Odd-expressing pericardial cells (arrowheads in C, D), whereas embryos carrying the larger deletion exhibited a reduced number of Odd-positive cells (arrows in B). Asterisks in A and D demarcate Odd expression in the gut. Q) The numbers of Odd-positive cells in each *Iro-C* mutant were statistically tested for significant differences from wild-type embryos using the Mann-Whitney test (* p < 0.01, ** p < 0.001, *** p < 0.0001). Odd-positive cells that are part of the lymph glands were not included in the counting. (E-H) *Iro-C* mutants exhibit an abnormal pattern of Tin-expressing cells. The brackets highlight some hemisegments in which the normal pattern of four Tin-positive myocardial cells and two Tin-expressing pericardial cells is disorganized. The arrowheads in G point to Tin-positive cells that are detached from the heart. (I-L) Phenotypes for Dmef2 varied in their severity and included loss of Dmef2-positive cells (arrows) as well as misalignment of the two myocardial cell rows (arrowheads). (M-P) Loss of *Iro-C* results in a reduction of Eve clusters. (R-T) Quantification of the phenotypes observed. The bar graphs depict the percentage of embryos that exhibited a particular phenotype for the indicated marker. All embryos are oriented with the anterior to the left. Dorsal views of embryos at stages 14-16 are shown in A-L. Lateral views of embryos at stages 10/11 are shown in M-P.

**Figure 7:**
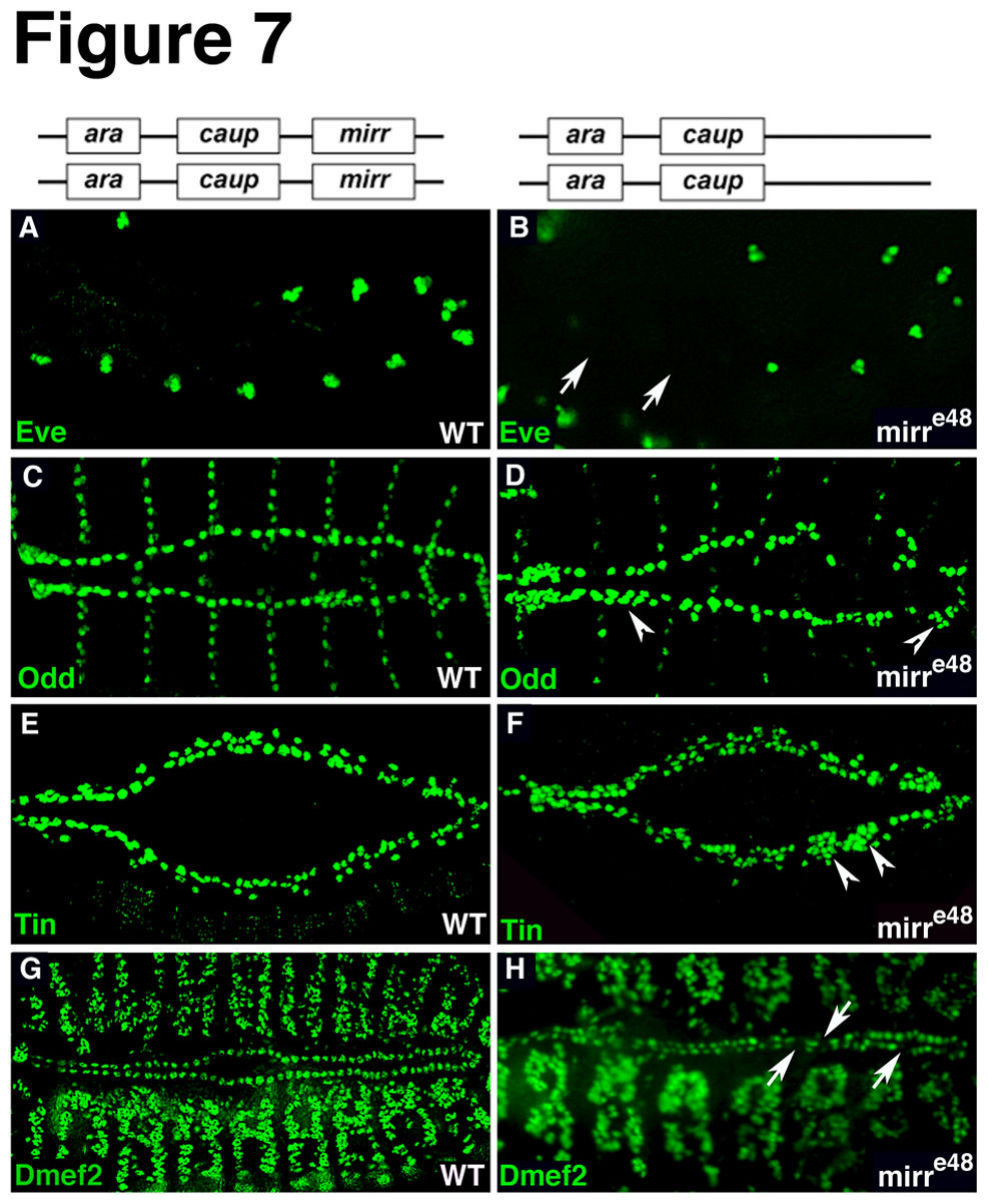
Heart phenotypes in embryos mutant for *mirr*. (A, B) *mirr* mutants have a reduced number of Eve clusters (arrows). (C, D) The number of Odd-expressing pericardial cells is increased in *mirr* mutants (arrowheads). (E, F) *mirr* mutants are characterized by an increased number of Tin-positive cells (arrowheads). (G, H) Myocardial Dmef2-expressing cells are slightly reduced in *mirr* mutants (arrows). Quantification of the phenotypes is included in the bar graphs shown in Figure 6 Q-T.

**Figure 8:**
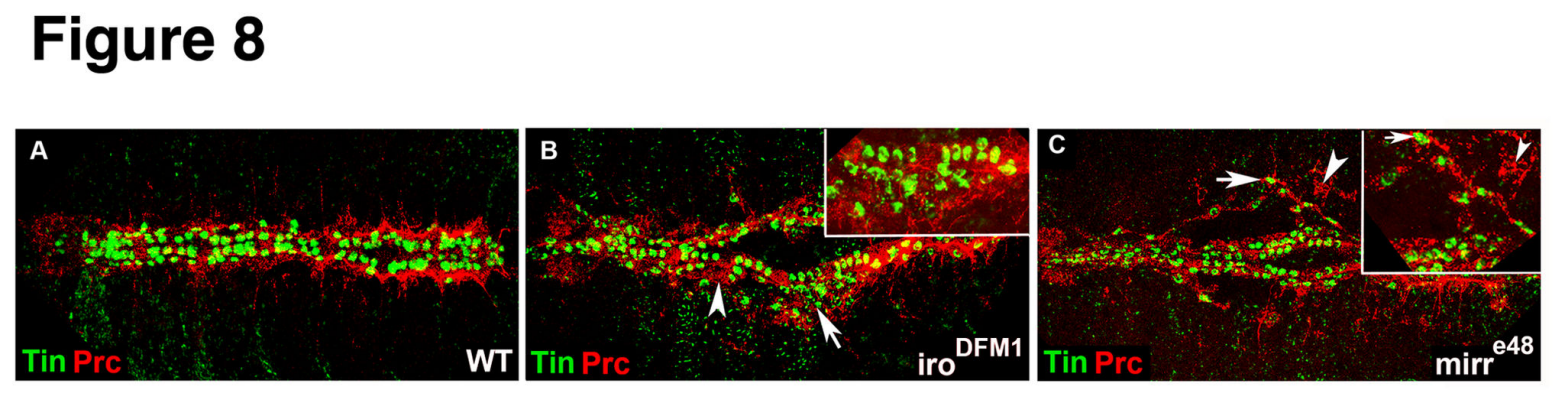
Double immunostainings for Tin and the pericardial marker Pericardin (Prc) confirm heart phenotypes affecting pericardial cells in *iro* ^DFM1^ and *mirr* ^*e48*^ embryos. (A) Wild-type expression of Tin and Prc at stage 16. (B) Prc expression is expanded in *iro* ^DFM1^ embryos (arrowhead) and includes additional Tin-positive cells (arrow). The inset is a higher magnification of the area around the arrow. (C) A *mirr* ^*e48*^ embryo showing the detachment phenotype of Tin-expressing pericardial cells (arrows) and ectopic Prc expression (arrowheads).

Since the analysis of embryos that are mutant for either the whole *Iro-C* (*Df(3L*)*iro-2*), for *ara/caup* only (*iro* ^*DFM1*^) or for *mirr* only (*mirr* ^*e48*^) revealed interesting phenotypes for crucial heart markers at early stages when the heart cells become specified, we wanted to determine how the early phenotypes affect heart formation at later stages. At stage 16, *iro* ^DFM1^ and *Df*(*3L*)*iro-2*/*iro* ^*DFM1*^ mutants appeared to have additional Odd-expressing pericardial cells (Figure 6A, C, D) and a statistical analysis confirmed this observation (Figure 6Q). Of note, the bar graphs in Figure 6Q-T include the quantification of heart phenotypes analyzed in *mirr* mutants shown in Figure 7. Embryos mutant for *mirr* were also characterized by an increase in Odd-expressing pericardial cells (Figure 7C, D). In contrast, *Df*(*3L*)*iro-2* embryos exhibited a reduced number of Odd-positive pericardial cells (Figure 6A, B). Immunostainings for Tin revealed an abnormal expression pattern at stage 14/15 (Figure 6E-H, R and Figure 7E, F). In wild-type embryos Tin is expressed in four out of the six myocardial cells in each hemisegment and in four pericardial cells (not all of which are always nicely detected in immunostainings before stage 16). As observed in *iro* ^DFM1^ and *mirr* ^*e48*^ embryos at stage 11/12, these embryos were also characterized by the presence of additional Tin-positive cells at stage 14/15 (Figure 6R). A smaller percentage of *iro* ^DFM1^ and *mirr* ^*e48*^ embryos had small gaps and some embryos exhibited both phenotypes, gaps and additional cells (denoted “mixed” in the histogram). Embryos of the deficiency *Df*(*3L*)*iro-2* exhibited either gaps or a combination of gaps in some hemisegments and accumulation of Tin-positive cells in other hemisegments (denoted “mixed” in the histogram). An additional phenotype observed in approximately 30% of *iro* ^DFM1^ and *mirr* ^*e48*^ embryos (not included in the histogram) indicates a detachment of Tin-positive pericardial cells (Figure 6G and Figure 8). To determine whether the additional Tin-positive cells are pericardial cells, a double immunostaining for Tin and Prc was performed. Indeed, Prc expression is detected around the additional Tin-positive cells (Figure 8A-C). As to the myocardial marker Dmef2, the phenotype encompassed the loss of generally a few Dmef2-expressing cells and a disorganization of the two myocardial cell rows (Figure 6I-L, S). A “disorganized” phenotype is characterized by small gaps and apparently additional cells as seen for example in the embryo shown in Figure 6K. Since the number of Dmef2 cells was not significantly changed in embryos with a “disorganized Dmef2 phenotype” (101 cells in *iro* ^DFM1^ embryos versus 104 cells in wild-type embryos), we conclude that this is simply a misalignment of the two myocardial cell rows. The majority of embryos mutant for *mirr* were characterized by a mild loss of Dmef2-expressing myocardial cells and did not exhibit defects in the alignment of the two myocardial cell rows (Figure 7G, H). It should be noted that embryos that carry the smaller deletion of the whole *Iro-C* (*iro* ^*DFM3*^) did not exhibit dramatic heart phenotypes at late stages. We also analyzed the phenotype for Eve at stage 10/11. At this stage Eve is expressed in 11 cell clusters that harbor precursors of Eve-positive pericardial cells and dorsal somatic muscles. In general, embryos of each investigated mutant background were characterized by a loss of Eve cell clusters (Figure 6M-P). Since the phenotypes differed in their severity, they were categorized according to the number of Eve cell clusters that were missing in the different mutants (Figure 6T). A comparison of the phenotypes shows that of all *Iro-C* factors *mirr* has the least impact on Eve-expressing cells. Only 15% of *mirr* mutants showed a phenotype for Eve (Figure 7A, B) and only up to three Eve cell clusters were missing. In contrast, 40% of the embryos mutant for *ara*/*caup* (*iro* ^*DFM1*^) exhibited a phenotype with approximately 15% of the embryos missing more than 6 Eve cell clusters. Taken together these results demonstrate that the loss of *Iro-C* affects heart formation at least in part by interfering with the normal diversification of heart cell types.

### Mesodermal overexpression of Iro-C elicits different effects on heart formation

Finally, we analyzed changes in the expression of heart markers after overexpressing each factor throughout the mesoderm. Generally, the percentage of embryos exhibiting a phenotype after overexpressing UAS-*ara* were weaker compared to the effects elicited by overexpressing UAS-*caup*. We cannot exclude that the difference in the severity of effects may be due to different expression levels of the cDNA constructs. Since *ara/caup* mutants are characterized by a loss of Eve-expressing cells we expected the opposite effect when overexpressing UAS-*ara* or UAS-*caup*. However, overexpression of either factor also resulted mainly in a reduction of Eve-positive cells in approximately 30% of the embryos (n = 150) (Figure S3A-C). Consistent with the results obtained from the analyses of *ara/caup* and *mirr* mutants, the effect of overexpressing UAS-*mirr* on Eve was only observed in 14% (n = 43) of the embryos and less severe compared to the effects elicited by UAS-*ara* or UAS-*caup* (Figure S3D). Consistent with the Eve phenotype analyzed in *mirr* mutants, this finding indicates that *mirr* has a lower impact on Eve expression as compared to *ara/caup*. Analyses of Odd-expressing pericardial cells revealed phenotypes that are complementary to the phenotypes observed in *ara/caup* and *mirr* mutants. Whereas the mutants were characterized by the presence of additional Odd-pericardial cells, overexpression of UAS-*caup* or UAS-*mirr* resulted in the loss of Odd-pericardial cells (Figure S3E, G, H). As to Tin, the phenotype is a dramatic disorganization after overexpression of either UAS-*caup* or UAS-*mirr* (Figure S3I, K, L). Interestingly, overexpression of either factor resulted in an ectopic accumulation of Tin-positive cells in the anterior part of the heart where the lymph glands are located.

In summary, our results add the *Iro-C* to the network of factors that play a role in dorsal mesoderm patterning. Our comprehensive analyses of changes in expression of heart marker genes demonstrate that all three members of the *Iro-C* are required for normal heart development, although their exact function remains to be studied in more detail. Moreover, the finding that *pnr* expression is unchanged yet Tin, Doc and Tup expression is not maintained at the normal level is an interesting finding suggesting that Pnr may only fully function in the presence of *Iro-C*.

## Discussion

Tissue patterning requires the spatial and temporal coordinated action of signals providing instructive or permissive cues that result in the specification of different cell types and their subsequent differentiation into different lineages. Our analyses of *Iro-C* deficient embryos demonstrate that *ara/caup* and *mirr* are required in the dorsal mesoderm for normal heart development. The heart phenotypes could be caused by alterations of the fine balance of the interactions between factors of the cardiac signaling network. In early stage *Drosophila* embryos the mesoderm is patterned along the anterior-posterior (AP) axis with cardiac and somatic mesodermal domains alternating with visceral mesodermal domains. The *tin*-positive mesoderm is specified as cardiac and somatic mesoderm under the influence of combined Dpp and Wg signaling [3,11,57]. Subsequently, the cardiac and somatic mesodermal domains are further subdivided by the action of the Notch pathway and MAPK signaling activated by EGFR and FGFR [6,7,58,59]. The Eve-expressing cell clusters that give rise to pericardial and DA1 somatic muscle cells [44,49], as well as the Doc expression pattern, distinguish the cardiac and somatic mesodermal domain from the visceral mesodermal domain [18]. The early expression pattern of Ara/Caup and Mirr at stages 10/11 suggests a role for *Iro-C* in patterning the dorsal mesoderm along the AP axis. Consistent with their previously described functions in other developmental contexts, members of the *Iro-C* may integrate signaling inputs and interact with other transcription factors to specify different dorsal mesodermal derivatives. Activation of the *Iro-C* by the EGFR pathway is required for the specification of the notum [60,61]. *Mirr* was shown to interpret EGFR signaling by eliciting a specific cellular response required for patterning the follicular epithelium [62]. During *Drosophila* eye development, *mirr* expression can be regulated by Unpaired, a ligand that activates JAK/Stat signaling [63]. In fact, the JAK/Stat signaling pathway has only recently been added to the signaling pathways that function in *Drosophila* cardiogenesis [64]. In chromatin immunoprecipitation experiments performed by Johnson et al. [64] *caup* was identified as a target of Stat92E, which is the sole transcriptional effector of the JAK/Stat signaling pathway in *Drosophila* [64,65]. Interestingly, the increase of Odd-pericardial cells and the additional Tin-expressing cells that were the characteristic phenotypes in *ara/caup* (*iro* ^*DFM1*^) and in *mirr* (*mirr* ^*e48*^) mutants are highly similar to the phenotypes in *stat92E* mutants described by Johnson et al. [64]. Also, as described for *stat92E* mutants, we noticed cell adhesion defects in a number of embryos as determined by the distant location of some Tin-expressing cells from the forming heart tube. As for establishing a possible link between JAK/Stat and *Iro-C* in the dorsal mesoderm and specifically in cardiogenesis, it would be necessary to determine for example whether *caup* and *mirr* can rescue the heart phenotype of *stat92E* mutants. Also, it would be interesting to compare the expression of the other crucial heart marker genes, Tup, Doc and Pnr, in *stat92E* mutants at early stages to determine to what extent the phenotypes of embryos mutant for *Iro-C* and for JAK/Stat signaling are similar.

Members of the *Iro-C* were shown to be positively or negatively regulated by signaling pathways that play crucial roles in heart development. Conversely, the *Iro-C* factors can also regulate the activity of at least one of these pathways. Specifically, Ara/Caup, as well as Mirr were shown to regulate the expression of the glycosyltransferase *fringe* and as a consequence modulate Notch signaling activity in the eye [25,63,66,67]. In the dorsal mesoderm, the lateral inhibitory function of Notch signaling establishes the proper number of heart and muscle progenitors [7,68]. Given the fact that *Iro-C* can regulate Notch activity it may be that the loss of *Iro-C* leads to an imbalance of progenitor cell specification resulting in an abnormal number of heart cells. Further studies are required to decipher the molecular mechanism by which *Iro-C* could integrate diverse signaling inputs and thereby function in the specification and differentiation of the different dorsal mesodermal derivatives.

**Figure 9:**
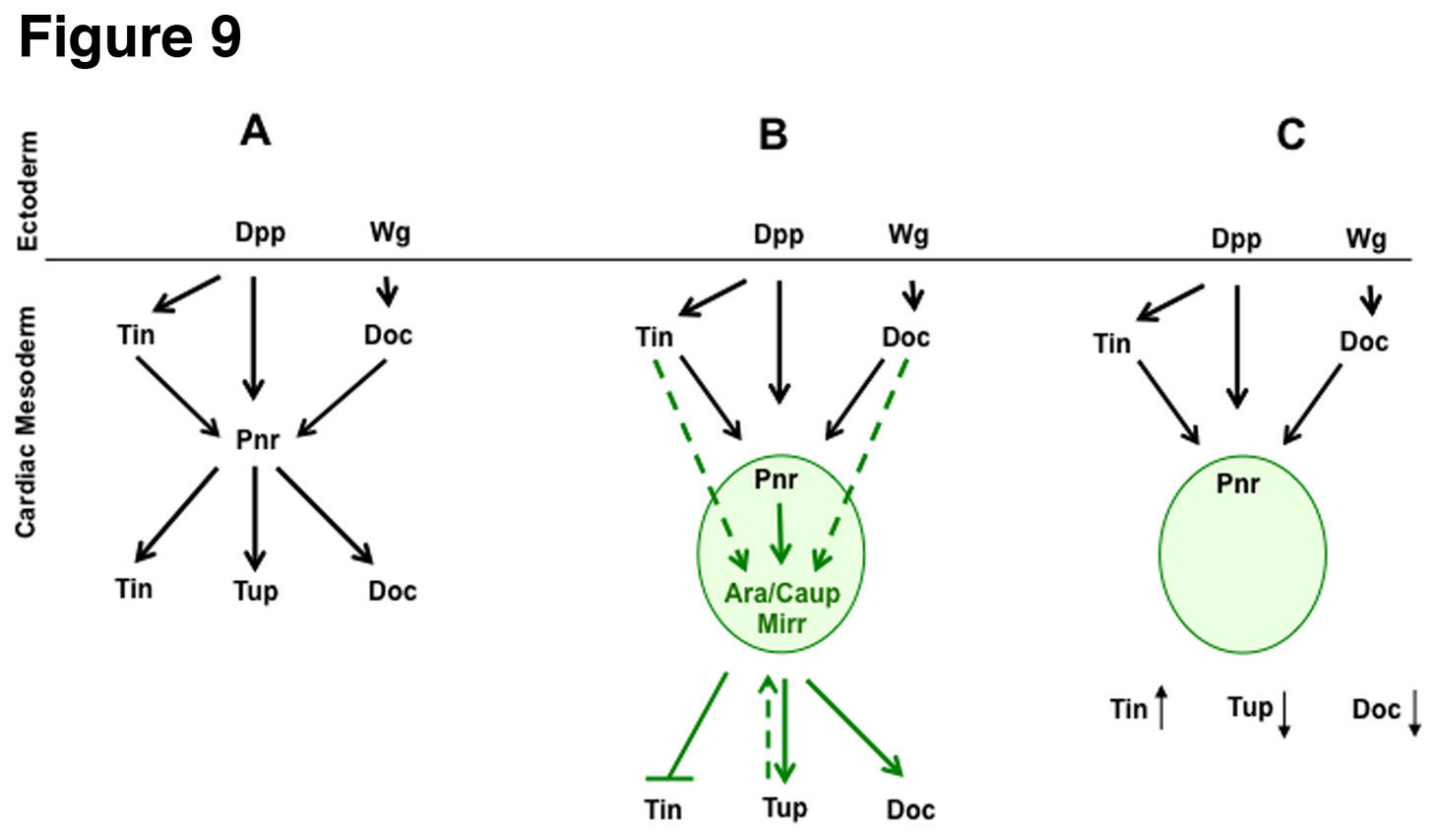
The cartoons depict regulatory interactions between the cardiac transcription factors including the members of the *Iro-C* based on our initial analyses on their function in the early dorsal mesoderm. For the sake of simplicity the cartoons neglect, for example, the different impacts of Ara/Caup and Mirr on the expression of the tested cardiac marker genes as well as additional regulatory interactions between the previously characterized factors. (A) In the previous model *pnr* was shown to be required for the maintenance of Doc, Tin and Tup in the cardiac mesoderm during stage 11. (B) Our data show that *ara/caup* and *mirr* act downstream of *pnr* or in combination with Pnr protein to regulate cardiac gene expression (specifically, to maintain Tup and Doc expression). Since *Iro-C* mutants were characterized by the presence of additional Tin-expressing cells, it appears that *Iro-C* is normally required to restrict the number of Tin-positive cells at stages 11/12. (C) In the absence of *Iro-C* Pnr is not sufficient to maintain Tup and Doc expression. The fact that we observe additional Tin-expressing cells could result from an earlier *pnr*-independent event in which *Iro-C* is involved. (A-C) Black arrows depict known interactions, green arrows depict our results, dashed arrows indicate the possibility that Tin, Doc and Tup could regulate Ara/Caup and Mirr directly without the involvement of Pnr.

To determine whether *Iro-C* can be positioned into the early transcriptional network that determines a cardiac lineage, we investigated the interdependency between crucial cardiac factors and *Iro-C* during cardiogenesis. Analyses of the expression of Ara/Caup and Mirr in *tin* ^346^, *Df*(*3L*)*DocA*, *pnr* ^VX6^ and *tup* ^*isl-1*^ embryos demonstrated the dependency of Ara/Caup and Mirr on all four factors. The strongest loss of Ara/Caup and Mirr expression was observed in *tin* ^346^ and *Df*(*3L*)*DocA* mutants, which clearly places *tin* and *Doc* upstream of Ara/Caup and Mirr. In *tup* ^*isl-1*^ and in *pnr* ^VX6^ mutant embryos, Ara/Caup and Mirr were strongly downregulated, however regarding Ara/Caup, some expression remained in segmental patches suggesting a different level of regulation. The currently available data indicates a positive and a negative regulatory effect of *pnr* on *Iro-C*. Whereas *pnr* restricts *Iro-C* expression in the dorsal ectoderm and in the wing disc, there is also evidence that *pnr* can positively regulate *Iro-C* in the wing disc [26,37]. Whether Pnr activates or represses *Iro-C* appears to depend on the presence of U-shaped (Ush), a protein that modulates the transcriptional activity of Pnr [37,69]. In the wing disc it was shown that an *Iro-C*-lacZ (IroRE^2^-lacZ) construct was activated in cells that contained Pnr but were devoid of Ush [37]. Our data demonstrate that in the dorsal mesoderm, the expression of Ara/Caup and Mirr depends on *pnr*. Additionally our analyses show that *pnr* expression is independent of *Iro-C*, an observation that was previously mentioned by Herranz and Morata [70]. This finding is intriguing with respect to the downregulation of Tup and Doc in *Iro-C* mutants. Pnr is required for the maintenance of Doc and for the initiation and/or maintenance of Tup [18,19]. Since *Iro-C* mutants exhibit a reduction in Doc-positive cells despite the presence of *pnr*, members of the *Iro-C* appear to be required independently or in addition to *pnr* to maintain expression of Doc. This could be investigated by expressing *ara*, *caup* and/or *mirr* in the mesoderm of *pnr* mutants to determine whether these factors are able to restore Doc expression. Alternatively, it may be that *Iro-C* is required indirectly meaning that its main function is to provide a molecular context in which Pnr can be active. For example, it is known that Ush can bind to Pnr thereby inactivating Pnr function [69]. It is conceivable that the absence of *Iro-C* affects the spatial expression of Ush. If, in the absence of *Iro-C*, the expression domain of Ush shifts into the Pnr expression domain, Ush could bind to Pnr and inactivate it in the region where Pnr is required to maintain the expression of Tup and Doc. Adding to the complexity of the interpretation of the observed phenotypes is our finding that the majority of embryos that are mutant for *ara/caup* or for *mirr* were characterized by supernumerary Tin-positive cells in the cardiac region by stage 11/12. This phenotype could still be observed at later stages when the heart tube forms. The additional Tin-positive cells are pericardial cells as determined by the expression of Prc around the Tin-expressing cells. Also, we did not observe an increase of Dmef2-positive myocardial cells. Hence, our data suggests a different level of regulation of Tin by the *Iro-C*. Similar to the findings of Johnson et al. [64], it may be that *Iro-C* is normally required to restrict Tin expression at an early stage. The regulation of Tin expression can be divided into four phases [64,71]. The phenotype we observe occurs when Tin expression becomes restricted to the myo- and pericardial cells in the cardiac region. In summary, our data adds *Iro-C* to *tin*, *pnr*, *Doc* and *tup* whose concerted actions establish the cardiac domains in the dorsal mesoderm (Figure 9). Further studies are required to re-evaluate our current understanding of the interactions between factors of the cardiac transcriptional network.

According to the expression pattern of Ara/Caup and Mirr we can distinguish between an early and late role for these factors, the latter being a role in the differentiation of heart cells.

Our analyses of the expression of Ara/Caup and Mirr during embryogenesis led to the identification of hitherto unknown heart-associated cells. We detected seven pairs of Ara/Caup and Mirr expressing cells and seven pairs of Mirr only expressing cells located along the dorsal vessel. We did not detect a co-expression with any of the known pericardial cell markers. Because there are seven pairs of these cells segmentally arranged, it was tempting to speculate that these cells may function, for example, as additional attachment sites for the seven pairs of alary muscles. The alary muscles attach the heart to the dorsal epidermis and their extensions can be visualized by Prc. Due to the lack of markers little is known about the development of the alary muscles [72]. Previous work by LaBeau et al. [72] demonstrated that the alary muscles attach to the dorsal vessel in the vicinity of the Svp pericardial cells and, in addition, more laterally to one of two distinct locations on the body wall. Maybe it is the Mirr-positive cells that identify the more lateral locations. Clearly, a detailed analysis is needed to identify the function of the Ara/Caup- and Mirr- as well as Mirr-expressing cells that are positioned along the heart tube and whose existence has now been revealed. Additionally, we identified on each side at the anterior end of the dorsal vessel four pericardial cells that co-express Ara/Caup and Eve. Their location at the anterior tip of the heart is intriguing. Further analysis is required to unambiguously determine whether these cells are, for example, the wing heart progenitor cells [73] or the newly identified heart anchoring cells [74]. It is also possible that they represent a yet undefined, novel subpopulation of pericardial cells. In any case, this finding suggests that Ara/Caup plays a role in the diversification of pericardial heart cell types. Future experiments aim to determine the developmental fate of these cells.

Taken together, our initial investigation of a role for *Iro-C* in heart development introduces *ara/caup* and *mirr* as additional components of the transcriptional network that acts in the dorsal mesoderm and as novel factors that function in the diversification of heart cell types.

Our results show that the role of the *Iro-C* and its individual members, respectively, appears to be rather complex and awaits in-depth analyses. Nevertheless, this work raises important questions regarding our current understanding of interactions between the well-characterized transcription factors that will be addressed in future studies.

## Supporting Information

Figure S1 **Ara/Caup and Mirr demarcate a novel heart or heart-associated cell type.** (A) Ara/Caup protein is not co-expressed with Tin. Arrows point to Ara/Caup expressing cells. (B) A double immunostaining for Mirr and Tin shows no co-expression of these factors. Arrows point to the pairs of Mirr-expressing cells that are adjacent to Tin-positive cells.
(TIF)

Figure S2 **Heart phenotypes in embryos mutant for the *Iro-C* (*iro*^*DFM3*^).** (A-C) Embryos lacking the three members of the *Iro-C* show a downregulation of Doc in the cardiac region (arrows). (D-F) Almost 80% of the *iro* ^DFM3^ embryos are characterized by additional Tin-expressing cells at stage 12 (arrowhead). The strong downregulation of FasIII (asterisk) demonstrates the impact of *Iro-C* on visceral mesoderm development. (G-I) *pnr* mRNA expression is unaffected in the majority (86%) of *iro* ^DFM3^ embryos. The arrows point to missing Eve cell clusters. (J-L) Loss of *Iro-C* results in a reduction of Eve cell clusters (arrow). (M-O) *iro* ^DFM3^ embryos were characterized by missing Odd-positive cells in some hemisegments (arrow) and additional Odd-expressing cells in other hemisegments (brackets). The overall number of Odd-expressing pericardial cells was not significantly changed compared to wild-type embryos.
(TIF)

Figure S3 **Heart phenotypes observed after mesodermal overexpression of individual members of the Iro-C.** (A, E, I) Wild-type expression of Eve (A), Odd (E) and Tin (I). (B, F, J) Overexpression of Ara results in (B) the loss of some Eve clusters, (F) a mild disorganization of Odd-expressing pericardial cells and (J) a mild disorganization of Tin-positive cells. The ectopic accumulation of Tin-expressing cells at the anterior end of the heart (arrowhead) appears to be in the region where the lymph glands are located (compare with (E) showing normal Odd expression in the lymph glands (lg)). (C, G, K) Overexpression of Caup not only leads to a loss of Eve clusters but also to a slight expansion of these clusters (C), (G) a dramatic loss of Odd-expressing pericardial cells and (K) a severe heart tube defect as can be seen by the disorganized arrangement of Tin-positive cells. (D, H, L) Embryos overexpressing Mirr are characterized by (D) a rather mild loss of Eve clusters, (H) a dramatic loss of Odd-expressing cells including pericardial and lymph gland (lg) cells and (L) accumulations of Tin-positive cells along the region where the heart tube forms.
In all images arrows point to missing cells whereas arrowheads point to additional cells or cell accumulation. Lateral views of stage 10/11 embryos are shown in A-D. Dorsal views of stage 16 embryos are shown in E-L.
(TIF)
