# Correction: The *Iroquois* Complex Is Required in the Dorsal Mesoderm to Ensure Normal Heart Development in *Drosophila*

**DOI:** 10.1371/annotation/e644be4d-7cda-417f-846a-723a52285c72

**Published:** 2013-11-07

**Authors:** Zhasmine Mirzoyan, Petra Pandur

The figure currently appearing as Figure 7 should be labeled as Figure 8, and the figure currently appearing as Figure 8 should be labeled as Figure 7. 

