# Supplementary figures and images for: The Iroquois Complex Is Required in the Dorsal Mesoderm to Ensure Normal Heart Development in Drosophila
Source: PLoS One. 2013 Sep 23;8(9):e76498. doi: 10.1371/journal.pone.0076498 (PMC3781054; doi:10.1371/journal.pone.0076498)

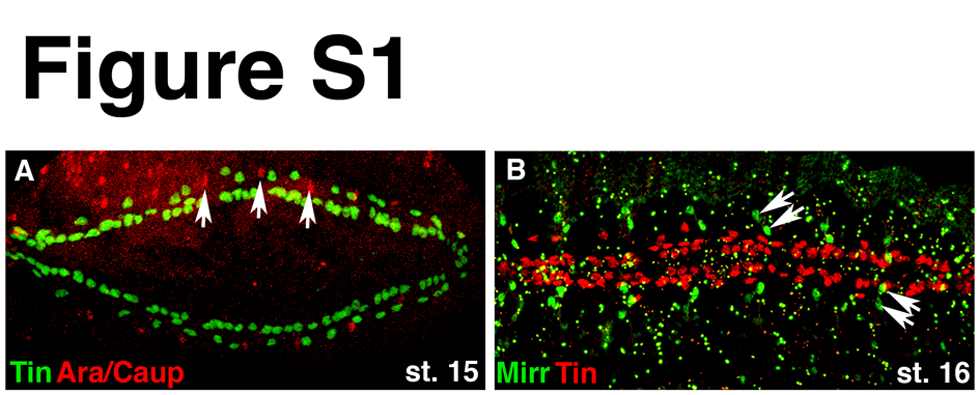

Supplement: Figure S1 — Ara/Caup and Mirr demarcate a novel heart or heart-associated cell type. (A) Ara/Caup protein is not co-expressed with Tin. Arrows point to Ara/Caup expressing cells. (B) A double immunostaining for Mirr and Tin shows no co-expression of these factors. Arrows point to the pairs of Mirr-expressing cells that are adjacent to Tin-positive cells. (TIF) [file pone.0076498.s001.tif]

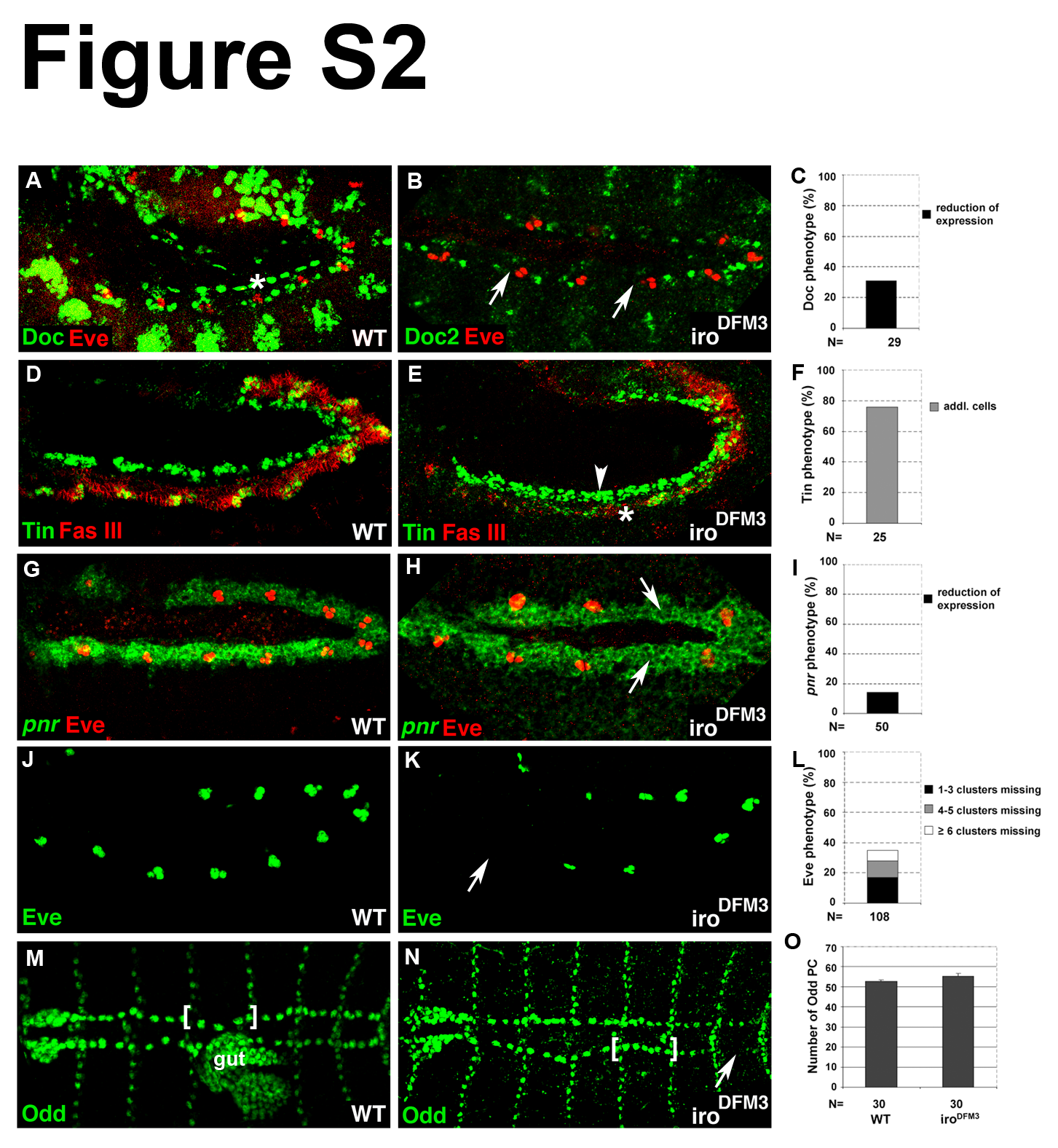

Supplement: Figure S2 — Heart phenotypes in embryos mutant for the Iro-C (iroDFM3). (A-C) Embryos lacking the three members of the Iro-C show a downregulation of Doc in the cardiac region (arrows). (D-F) Almost 80% of the iro DFM3 embryos are characterized by additional Tin-expressing cells at stage 12 (arrowhead). The strong downregulation of FasIII (asterisk) demonstrates the impact of Iro-C on visceral mesoderm development. (G-I) pnr mRNA expression is unaffected in the majority (86%) of iro DFM3 embryos. The arrows point to missing Eve cell clusters. (J-L) Loss of Iro-C results in a reduction of Eve cell clusters (arrow). (M-O) iro DFM3 embryos were characterized by missing Odd-positive cells in some hemisegments (arrow) and additional Odd-expressing cells in other hemisegments (brackets). The overall number of Odd-expressing pericardial cells was not significantly changed compared to wild-type embryos. (TIF) [file pone.0076498.s002.tif]

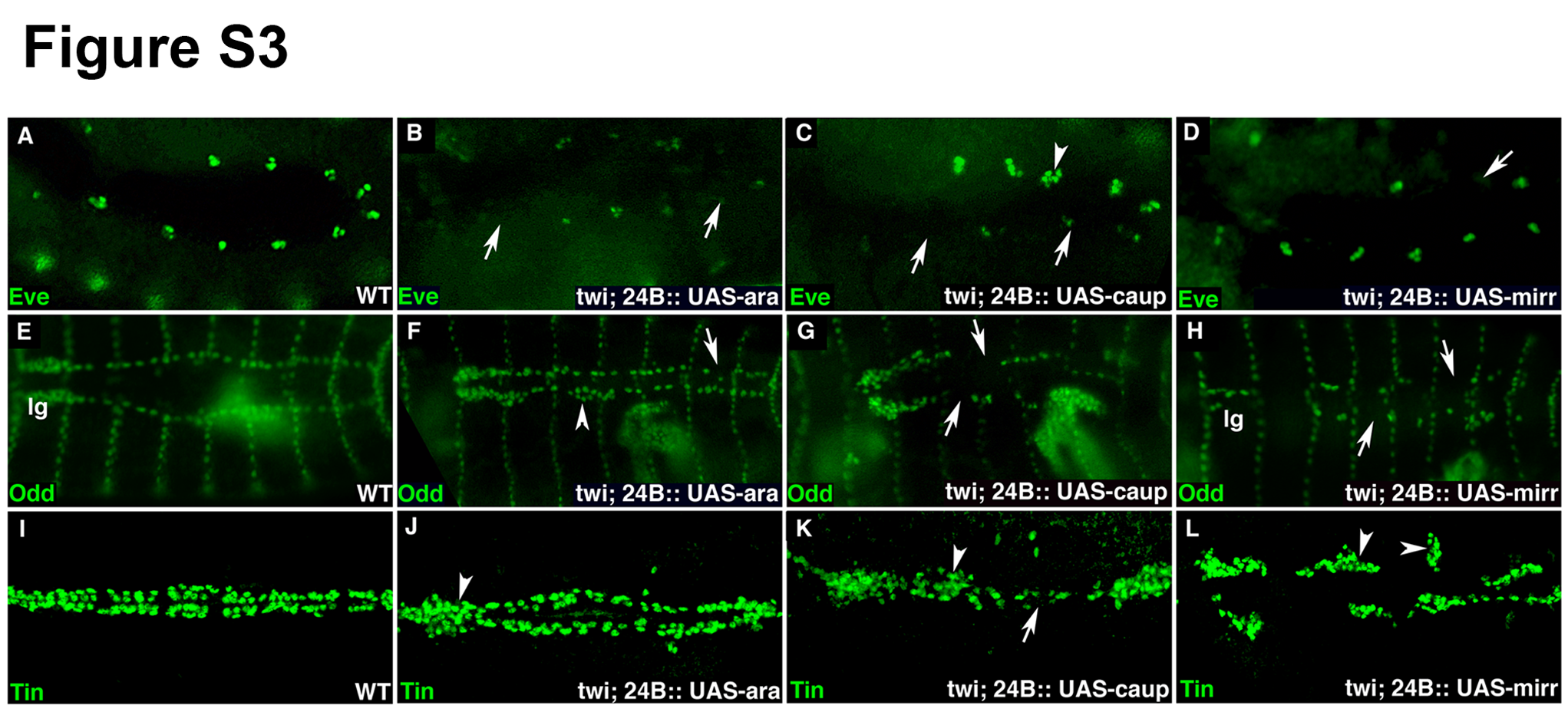

Supplement: Figure S3 — Heart phenotypes observed after mesodermal overexpression of individual members of the Iro-C. (A, E, I) Wild-type expression of Eve (A), Odd (E) and Tin (I). (B, F, J) Overexpression of Ara results in (B) the loss of some Eve clusters, (F) a mild disorganization of Odd-expressing pericardial cells and (J) a mild disorganization of Tin-positive cells. The ectopic accumulation of Tin-expressing cells at the anterior end of the heart (arrowhead) appears to be in the region where the lymph glands are located (compare with (E) showing normal Odd expression in the lymph glands (lg)). (C, G, K) Overexpression of Caup not only leads to a loss of Eve clusters but also to a slight expansion of these clusters (C), (G) a dramatic loss of Odd-expressing pericardial cells and (K) a severe heart tube defect as can be seen by the disorganized arrangement of Tin-positive cells. (D, H, L) Embryos overexpressing Mirr are characterized by (D) a rather mild loss of Eve clusters, (H) a dramatic loss of Odd-expressing cells including pericardial and lymph gland (lg) cells and (L) accumulations of Tin-positive cells along the region where the heart tube forms. In all images arrows point to missing cells whereas arrowheads point to additional cells or cell accumulation. Lateral views of stage 10/11 embryos are shown in A-D. Dorsal views of stage 16 embryos are shown in E-L. (TIF) [file pone.0076498.s003.tif]
